# Optimized wake-superposition approach for multiturbine wind farms

**DOI:** 10.1038/s41598-023-33165-4

**Published:** 2023-04-24

**Authors:** Deshun Li, Jixiang Chang, Gaosheng Ma, Chunyu Huo, Rennian Li

**Affiliations:** 1 College of Energy and Power Engineering, Lanzhou University of Technology, 287 LanGongPing Road, Qilihe District, Lanzhou, 730050 China; 2 Gansu Provincial Technology Centre for Wind Turbines, Lanzhou, 730050 China; 3 Gansu Provincial Key Laboratory of Fluid Machinery and Systems, Lanzhou, 730050 China

**Keywords:** Renewable energy, Wind energy

## Abstract

Optimizing the wind farm layout requires accurately quantifying the wind-turbine wake distribution to minimize interference between wakes. Thus, the accuracy of wind turbine wake superposition models is critical. The sum of squares (SS) model is currently touted as the most accurate, but its application in engineering is hampered by its overestimation of the velocity deficit of the mixed wake. Therefore, previous work relied on approximate power calculations for performing optimization. The physical meaning of the SS model is unclear, which makes optimization difficult. In this study, a univariate linear correction idea is proposed based on the linear increase phenomenon of the SS method error. The unknown coefficients are obtained by fitting experimental data. The results demonstrate that the proposed method can accurately quantify the full-wake two-dimensional distribution of the mixed wake.

## Introduction

Many countries are actively developing wind energy, which, as a renewable energy source, is expected to solve the energy crisis. Wind turbines convert wind energy into electrical energy through the interaction between the wind and blades. Incoming flow passing through the wind turbine creates a wake area with reduced wind velocity, which exchanges energy with the surrounding air and gradually returns to calm conditions as the wake develops. When the downstream wind turbine is in the wake region of the upstream wind turbine, its production capacity will be reduced, which can reduce annual power generation by as much as 10–20%^1^. Therefore, studying wake interference between wind turbines is of great significance in site selection, arranging wind turbines, and predicting power from wind farms. The CFD method of solving the N-S equation can accurately simulate the wake effect. However, it requires numerous costly computations and is difficult to apply in complex environments. Therefore, convenient and concise analytical wake models are popular in the wind industry. Therefore, this study proposes an optimized superposition model for wind Turbines, which can better meet the urgent needs of the industry.

In 1986, Jensen^2^ derived the Park one-dimensional (1D) model based on the conservation of momentum. Building upon it, Tian^3^ and Zhang^4^ both used a cosine-shape function to redistribute the wake deficit in the lateral direction and an extended two-dimensional (2D) model. Yang^5,6^ then proposed the wake model of Gaussian distribution and quadratic polynomial distribution (Park–Gauss model and Park–polynomial model), which can accurately simulate the far wake. The above models utilize the Park model to solve the 1D wake and then apply a specified equation to redistribute the wake. Ge^7^ argued that this violated the local mass conservation and proposed a 2D wake model that directly extends the Jensen model. Due to the complete obstruction of free flow in the center of the rotor, the near wake has a “W” shape^8^. To solve this problem, Keane^9,10^ proposed a full wake model based on the double Gaussian assumption of the wake distribution, but it had the worst agreement with the LES data compared to other models^11^. Later, some scholars^12–14^ proposed a wake model for the yaw condition.

The above research involves the wake model of a single wind turbine. In practical engineering, the wake interference between multiple wind turbines needs to be considered, and the superposition of wake models often simulates the actual wake situation. The commonly used wake superposition methods^15^ are the geometric sum (GS), linear superposition (LS), energy balance (EB), and the sum of squares of velocity deficits (SS). LS, proposed by Lissaman^16^, assumes that the velocity deficit of a mixed wake is equal to the sum of the velocity deficits of each upstream unit. Crespo^17^ found that the LS method overestimated the velocity deficit of the mixed wake and even had a negative velocity value. ES is an energy conservation model based on a simplified energy equation, which assumes that the sum of the kinetic energy losses of the incoming flow after passing through each upstream unit is equal to the kinetic energy loss in the mixed-wake region. In contrast, SS, which is currently the most widely used superposition model, considers the velocity deficit in the mixed-wake area to be equal to the square root of the sum of the velocity deficits in the wake area of each upstream unit. Kuo^18^ identified SS as the most accurate among the methods. However, Chamorro^19^ found that the wake recovery speed of two wind turbines with an in-line setup is sometimes higher than that of a single wind turbine. Meanwhile, the SS method leads to a deficit in the mixed-wake velocity, which must be higher than that of the single unit. Accordingly, the overestimation of the wake-velocity deficit is unrealistic.

This study aims to address the problem of the SS method overestimating the velocity deficit of the far wake. As such, the SS method is linearly optimized, considering the thrust coefficient of the downstream wind turbine and the area ratio of the overlap with the wake region. Coefficient fitting is performed according to the wind-tunnel experimental data, and wind-turbine full-wake optimization is conceptualized.

## Experimental setup and results

**Figure 1:**
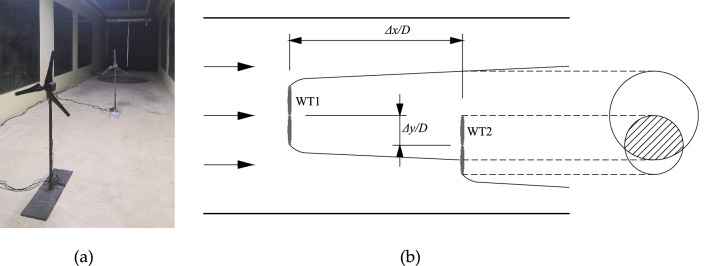
(**a**) Photograph of the experiment site, and (**b**) schematic of relative position of upstream and downstream wind turbines.

Wind tunnel experiments were conducted for different turbine layouts to acquire support data for the present study. The wind tunnel was 15 m long, 2 m wide, and 2 m high; the design wind velocity was 1–20 m/s; and the wind velocity reduction per meter along the flow direction was $u_{loss} = 0.009u_{\infty }$. In the experiments (Fig. 1a), two wind turbine models with rotor diameters *D* of 0.44 m and hub height of 0.65 m were arranged in the wind tunnel, and the mean incoming wind speed was 6 m/s. The wind turbine blades are connected to a small generator by a rotating shaft, and the speed is controlled by t adjusting the load connected to the generator. The error of single wind turbine speed can be maintained within 10%, and both wind turbine blade tip speed ratios are controlled at 5.5. The axial wake velocity was acquired using a pitot tube mounted on a three-dimensional coordinate frame. The Pitot tube was arranged on a plane with a horizontal height equal to those of the wind turbine hub: a sampling frequency of 10 Hz and average of 45 s for each measurement point were utilized. Figure 1b shows the relative positions of the two wind turbines. Regarding their arrangement, three tandem cases (Case1–3) with a radial spacing of 0 and axial spacing *Δx/D* of 4, 6, and 8, and three staggered cases (Case 4–6) with *Δx/D* = 4 and radial spacing *Δy/D* of 0.3, 0.5, and 0.7, respectively, were considered.

**Figure 2:**
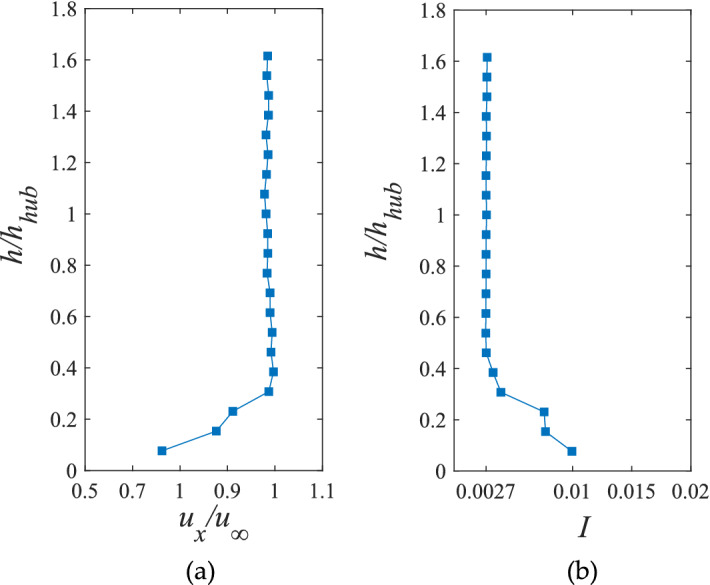
Normalized (**a**) velocity and (**b**) turbulence intensity vertical profiles.

Figure 2 shows the vertical profile of the normalized velocity and turbulence of the wind tunnel measured by the hot wire collection with a frequency of 1000 Hz. At 6 m/s incoming flow, there will be a velocity gradient of about 0.3 times the hub height of the wind turbine in the wind tunnel, but the velocity and turbulence fluctuations near the hub height are very small, and the incoming turbulence intensity is stable at 0.2%, so the incoming flow conditions meet the experimental requirements.

**Figure 3:**
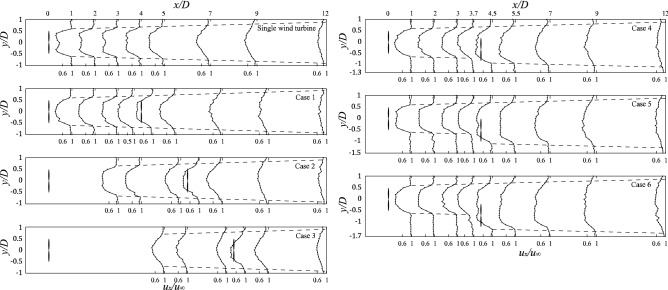
Normalized wind tunnel measurement results of single wind turbine and Cases 1–6 in the horizontal plane at the height of the hub center. The wake profile (dotted line) and the relative position of the wind turbines are marked on the figure.

Normalized measurements were obtained for a single wind turbine and Case 1–6 through wind tunnel experiments (Fig. 3**)**. The dashed line is the wake profile fitted with the wake velocity recovered to $99\% (u_{\infty } - x \cdot u_{loss} )$. Noticeably, the wake development behind the wind turbine is approximately a linear expansion (wake growth rate *k* = 0.025). The arrangement of the wind turbines does not affect the wake expansion, which is consistent with the linear-expansion assumption of the wake model. Comparing the single wind turbine with Case 1 reveals that the downstream placement of the wind turbine will lead to faster wake recovery due to the disturbance of the downstream wind turbines increasing the energy exchange between the mixed wake and surrounding atmosphere. Similarly, comparing Cases 4–6 reveals that the radial spacing increases as the wake is perturbed less, and the wake recovers slower.

## Wake modeling

### Single wake model

**Figure 4:**
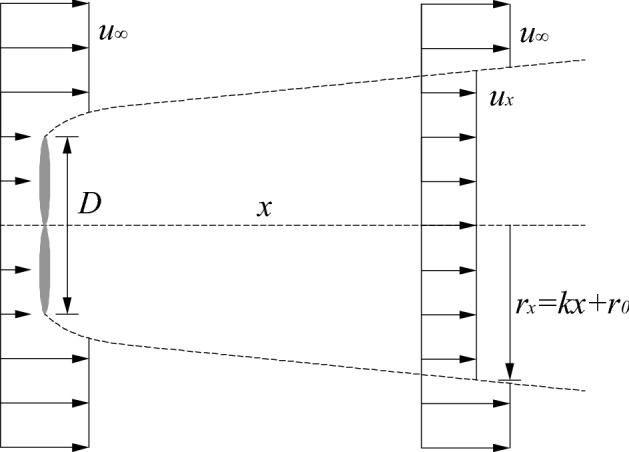
Schematic of the Park model.

The Park model developed by Jensen^2^ is the pioneering turbine-wake model derived from the conservation of mass by applying the Betz theory to relate the velocity deficit in the wake to the induction factor *a*. It has been extensively used in commercial software (e.g., WAsP, WindPRO, WindSim, Wind-Farmer, and OpenWind). The velocity deficit, which changes with the streamwise distance from the turbine rotor, is kept constant within the wake radius, so it is also called the “top-hat” model due to its shape, as shown in Fig. 4.

In this study, *x* denotes the streamwise distance from the turbine rotor of diameter *D*; $u_{\infty }$ is the mean incoming flow speed; and $u_{x}$ and *r*_*x*_ are the average wake velocity and the wake radius at a distance *x* from the turbine rotor, respectively. Linear growth of the wake radius with downwind distance is assumed for simplicity, which is verified by wind-tunnel measurements^20^ and LES numerical results^21^. The specific Park model formula is given by

1 $$u_{x} = u_{\infty } \left[ {1 - \frac{2a}{{\left( {1 + k \cdot x/r_{0} } \right)^{2} }}} \right],$$

where *k* is the wake growth rate, which is often taken as 0.1, as suggested by Jensen^2^. Different values of *k* were suggested in later studies^22,23^, such as 0.05 and 0.075 for offshore and onshore wind turbines, respectively. This study utilizes *k* = 0.025 based on the wind-tunnel experimental data. $r_{0}$ is the initial wake radius behind the turbine rotor. Due to factors such as tip vortex, $r_{0} > D/2$. According to actuator disc theory, the calculation formula is expressed as

2 $$r_{0} = 0.4d\sqrt {\frac{1 - a}{{1 - 2a}}} .$$

The top-hat distribution of the Park model tends to underestimate the velocity deficit in the wake center and overestimate it at the wake edges. To reasonably simulate the distribution of the velocity deficit in the wake using the Park model, a Gaussian function and a quadratic polynomial to describe the wake velocity (Park–Gauss model, Park–polynomial model) was introduced in the literature^5,6^, with good agreement with actual measurements being achieved. The wakes will be super positioned for multiple wind turbines based on these two 2D models.

The velocity deficit distribution in the near-wake cannot be simulated well because the wake model does not consider dominant factors, such as the hub and hub vortex. The experimental data in the near-wake exhibited a W-shaped distribution^8^. As the hub vortex developed, the velocity deficit at the hub height decreased, and the distribution gradually changed from a W-shaped to a Gaussian-shaped distribution.

### Wake superposition method

Wake interactions are not fully understood due to the complex turbulence phenomena within the mixed wake. Four semi-empirical formulas for determining the wake velocity of the downstream wind turbine are described in the literature^18^. Taking SS as the sum of the squared wake velocity deficits,

3 $$\left( {1 - \frac{{u_{i(x,r)} }}{{u_{\infty } }}} \right)^{2} = \sum\limits_{j = 1}^{N} {\left( {1 - \frac{{u_{i(x,r),j} }}{{u_{j(0,0)} }}} \right)^{2} } ,$$

where $u_{i(x,r)}$ is the wind velocity at position (*x*, *r*) within the wake of wind turbine *I*, $u_{i(x,r),j}$ is the wind speed at turbine *i* due to (the wake of) turbine *j*, and *u*_*j*(0,0)_ is the wind speed at wind turbine *j*.

**Figure 5:**
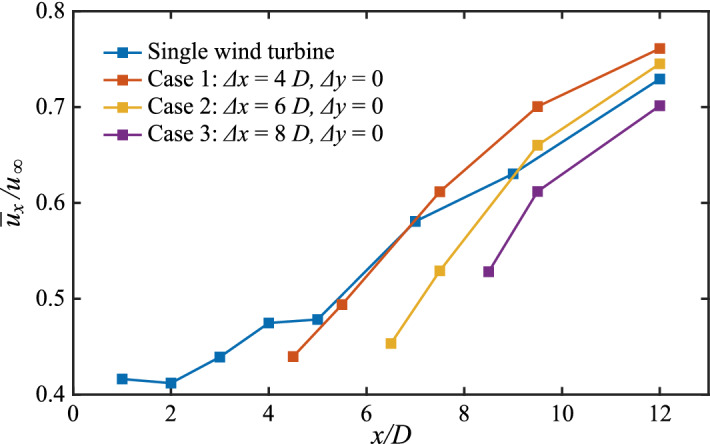
Distribution of the average wake velocity along the streamwise direction.

Experimental investigations of the wake interaction^24^ have demonstrated that SS is the most accurate of the four formulations, although it has no practical physical meaning. The single wake model ignores the wake rotational effect and turbulence, whereas the overlapping wake inevitably causes an increase in turbulent kinetic energy. The complex turbulent structure in the wake will accelerate the energy exchange between the wake and atmosphere, increasing the wake recovery. Figure 5 shows the distribution of mean wake velocity as wake develops for Cases 1–3 compared to single wind turbines. In the far wake, the wake velocity is greater for the axially spaced 4*D* and 6*D* cases than for the single wind turbine. The literature^19^ confirms that the presence of a wind turbine downstream will result in the mixed wake achieving a higher recovery velocity. The superposition principle of the SS method inevitably leads to an increase in the velocity deficit of the mixed wake, thereby overestimating the velocity deficit after superposition. However, realizing improvement from the experimental data is difficult because the physical basis of SS needs clarification^18^.

**Figure 6:**
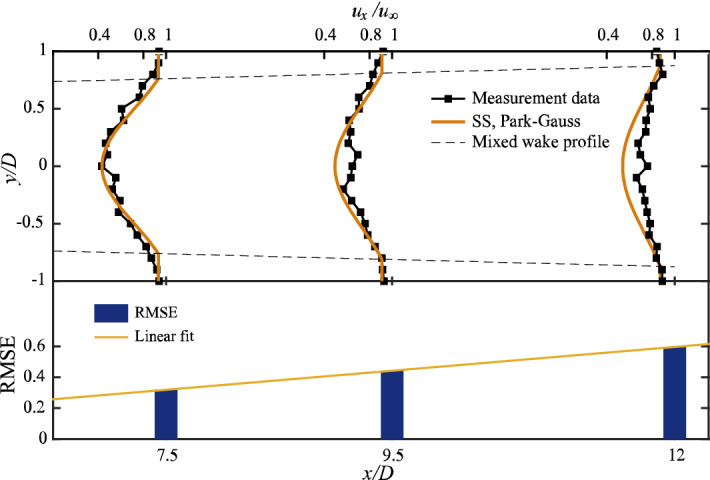
Results of model speed compared to measured values for Case 1 (other cases have the same pattern and are not shown here).

Figure 6 shows the results after superimposing the two Park–Gauss models with *Δx* = 4*D* using the SS method. Because the region between 4 and 6*D* is affected by the downstream wind turbine central vortex, 7.5*D*, 9.5*D*, and 12*D* are selected here for comparative analysis. The root mean square error (RMSE) is introduced in this study to measure the accuracy of the model compared to the experimental values:

4 $${\text{RMSE}} = \sqrt {\frac{{\sum\limits_{i = 1}^{n} {\left( {u_{i} - u_{i}^{{{\text{mea}}}} } \right)^{2} } }}{n}} ,$$

where $u_{2}^{\exp }$ and $u_{i}^{{{\text{mea}}}}$ are the model and measured velocity of measurement point *i*, respectively. n statistical measurement points are considered.

Figure 6 shows that the RMSE increases almost linearly with wake development. Therefore, the deviation of the downstream wind-turbine full wake from the actual value exhibits a linear pattern. As mentioned before, theoretically optimizing the SS is difficult. In this study, the above problem is reduced to a univariate linear optimization problem to obtain an accurate full wake model for the downstream wind turbine.

### Optimization of wake superposition method

Based on the assumption of a linear RMSE progression, the wake velocity $u_{i(x,r)}$ at different locations of the downstream wind turbine is considered the independent variable, and the optimized wake velocity $u_{i(x,r)}^{{{\text{opt}}}}$ is considered the dependent variable. They satisfy the following relationship:

5 $$u_{i(x,r)}^{{{\text{opt}}}} \sim \alpha \cdot (x/D) \cdot u_{i(x,r)} + \beta ,$$

where* α* and *β* are unknown parameters, which can be obtained by fitting experimental data. However, considering that the last wind turbine with a different arrangement will lead to different unknown parameters, the relationship equation is not universal. To solve the above problem, the thrust coefficient C_*T*_ is introduced in this study:

6 $$C_{T} = \frac{T}{{\frac{1}{2}\rho A_{x} u_{i(0,0)}^{2} }} = \frac{{u_{i(0,0)}^{2} - u_{i(x,r)}^{2} }}{{u_{i(0,0)}^{2} }},$$

where *T* is the thrust, *ρ* is the air density, and *A*_*x*_ is the cross-sectional area of the wake at a distance *x* from the rotor. A different position of the last wind turbine changes the value of the incoming flow velocity. Thus, the thrust coefficient of the downstream wind turbine can characterize the axial spacing variation. Moreover, as the radial spacing of the wind turbine changes, it will partly appear in the wake. The smaller the overlap area between the wake and wind turbine, the smaller the disturbance by the upstream wake, and the more accurate the mixed wake calculated by the SS method. Therefore, the thrust coefficient and overlap area ratio are applied to the relation:

7 $$u_{i(x,r)}^{{{\text{opt}}}} = \alpha \cdot C_{T} \cdot \left( {A_{mix} /\pi r_{2}^{2} } \right) \cdot (x/D) \cdot u_{i(x,r)} + \beta ,$$

where $A_{mix}$ is the area of overlap between the downstream wind turbine and the upstream wake (shaded area in Fig. 1b), and *r*_*2*_ is the radius of the downstream wind turbine.

**Figure 7:**
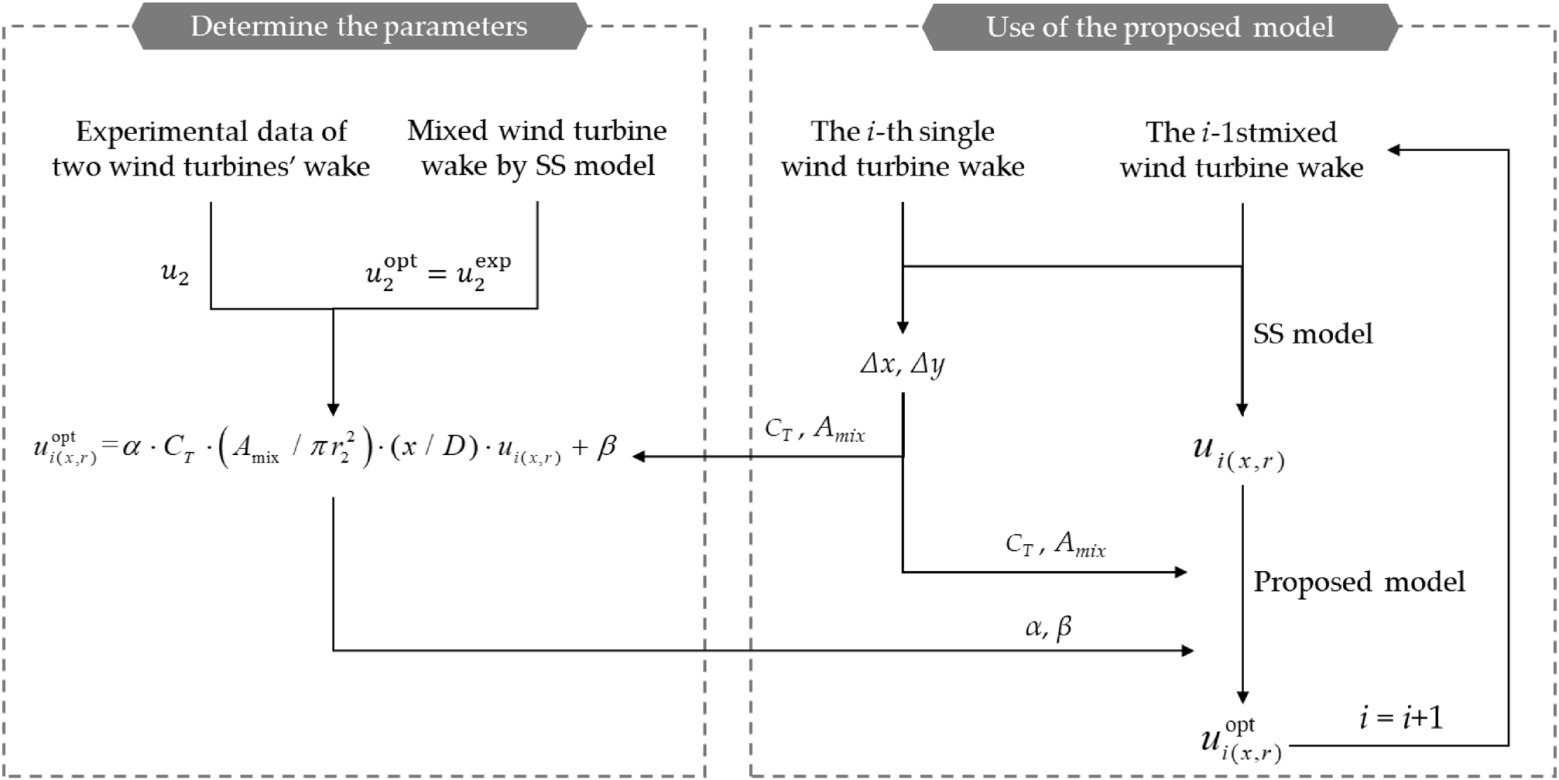
Flow chart of the correction method and the use of the proposed model.

Figure 7 shows the flow chart of the use of the proposed model, the model coefficients were obtained for different arrangements (Table 1) from the experimental values, where the two single wake models (Park–Gauss and Park–polynomial models) were separately superimposed. Therefore, after taking the mean value, *α* = 0.15 and *β* = 1.2 for the Park–Gauss model, and* α* = 0.16, *β* = 1.3 for the Park–polynomial model. From the data results in Table 1, it can be seen that the correction coefficients backwardly introduced by using the experimental values as the optimization objectives are applicable to different arrangements. On this basis, the mixed wake flow of the *i*-1st wind turbine and the single wake flow model of the *i*-th wind turbine are superimposed and the wake flow is corrected based on the determined parameters.

**Table 1:** Model coefficients under different arrangements.

Case	*Δx/D*	*Δy/D*	Park–Gauss		Park–polynomial	
			*α*	*β*	*α*	*β*
Case 1	4	0	0.1485	1.342	0.1568	1.4126
Case 2	6	0	0.1471	1.2513	0.1659	1.3576
Case 3	8	0	0.1661	1.1724	0.1739	1.2278
Case 4	4	0.3	0.1647	1.1921	0.1871	1.2906
Case 5	4	0.5	0.1387	1.2673	0.146	1.3282
Case 6	4	0.7	0.2079	1.2792	0.2219	1.3437

## Results and analysis

### Tandem layout

**Figure 8:**
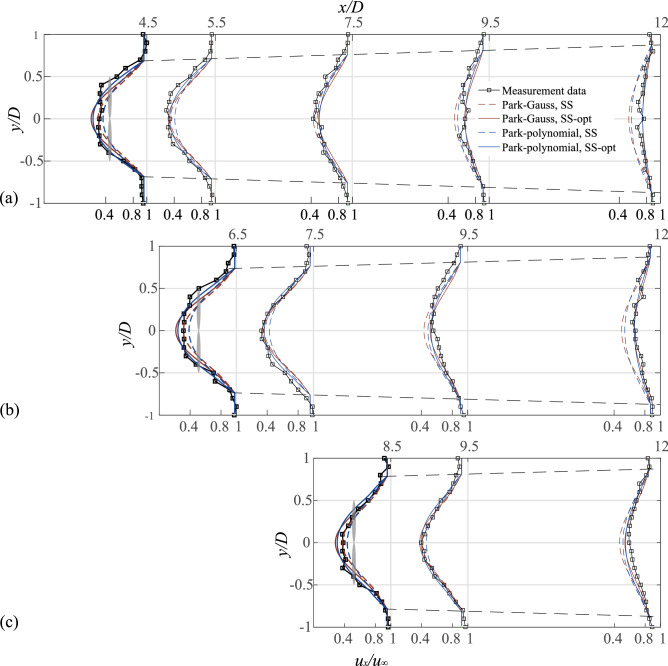
Normalized wake velocity deficit in the lateral direction for cases 1–3, (**a**)$\Delta x = 4D$, (**b**)$\Delta x = 6D$, (**c**)$\Delta x = 8D.$

**Figure 9:**
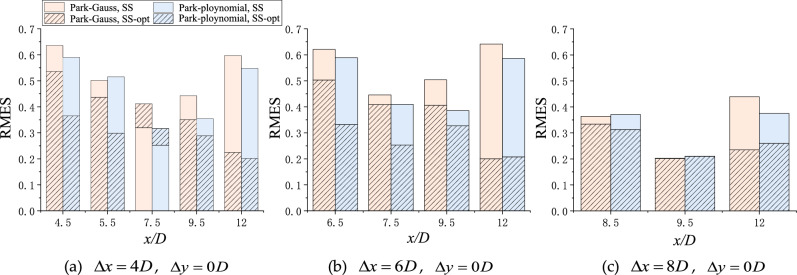
RMSE of the turbine-wake model from the experimental values in the tandem layout before and after optimization.

The tandem distribution (Cases 1–3) is simulated using the proposed superposition method, which is based on two two-dimensional single wake models (Park–Gauss and Park–polynomial models). As shown in Fig. 8, the dashed and solid lines denote the results from the SS and optimized models, respectively. The gray shading represents the relative positions of the downstream wind turbines. To quantify the error between the model and experiment, the RMSE corresponding to the two models is shown in Fig. 9. The specific analysis is as follows:

The velocity of the wake center recovered faster because it is affected by the hub vortex at the 0.5*D* position behind the downstream wind turbine, which results in the wake of this cross-section not fitting the Gaussian distribution. Therefore, the combination model cannot fit the experimental value at the center of the wake. The two models underestimated the velocity deficit of the wake in this cross-section, and the RMSE of the measured value was reduced after optimization.

In the case of *Δx* = 4*D* (Fig. 8a), the two models underestimated the wake deficit before 7.5*D* but overestimated the wake deficit in subsequent cross-sections. Therefore, the RMSE value first decreased and then increased with wake development. After optimization, the RMSE of the Park–Gauss and Park–polynomial models at the 12*D* cross-section were reduced from 0.597 and 0.548 to 0.225 and 0.202, respectively, implying that the model accuracy was greatly improved.

After optimization, the RMSE of the Park–polynomial model was found to be generally lower than that of the Park–Gauss model. Hence, the Park–polynomial model fitted better in the tandem layout.

### Staggered layout

**Figure 10:**
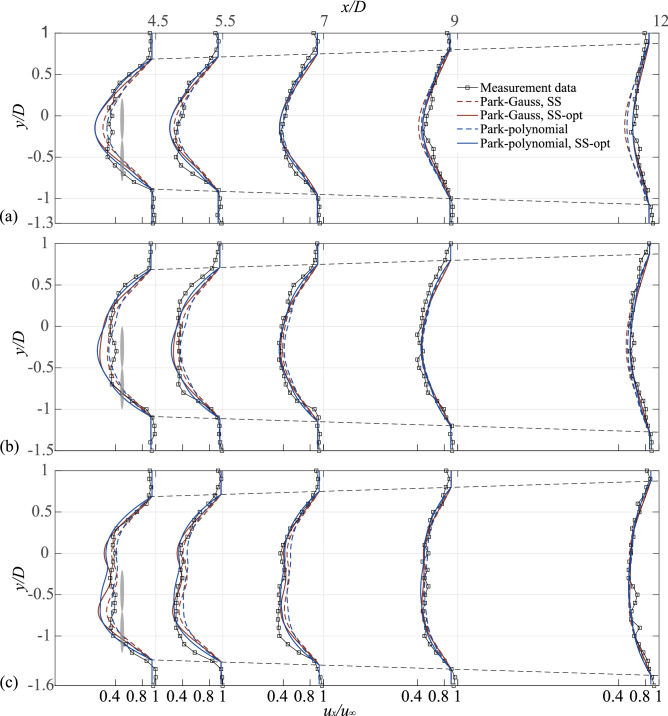
Normalized wake velocity deficit in the lateral direction for Cases 4–6, (**a**)$\Delta x = 4D$, $\Delta y = 0.3D$, (**b**)$\Delta x = 4D$, $\Delta y = 0.5D$, (c)$\Delta x = 4D$, $\Delta y = 0.7D.$

**Figure 11:**
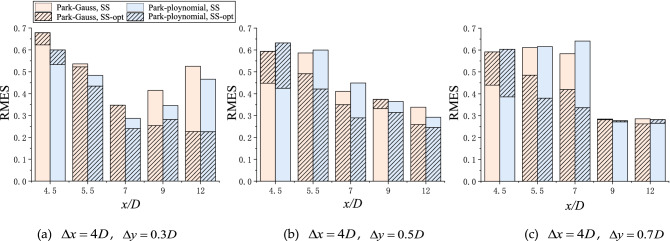
RMSE of the turbine-wake model from the experimental values in the staggered layout before and after optimization.

The same model fitting and error analysis as those above were performed for the staggered row arrangement (Cases 4–6). The specific analyses from Figs. 10 and 11 are as follows:

As the radial spacing *Δy* increased, the RMSE between the two models and the experimental values in the far-wake (9*D*, 12*D*) gradually decreased. At the 12*D* cross-section, the corresponding deviations in the Park–Gauss model for Cases 4–6 were 0.525, 0.338, and 0.286, respectively, and they were all reduced to approximately 0.26 after optimization.

Due to the hub vortex of the downstream wind turbine, the wake center velocity of the downstream wind turbine near-wake (at 0.5*D* and 1.5*D* cross-section from the downstream wind turbine) recovered quickly. Hence, the predictions from the wake model were not well-matched with the measurements, resulting in the RMSE of the optimized model increasing. The overall optimized RMSE decreased gradually as the wake developed.

The histogram (Fig. 9) shows that the corresponding RMSE of the Park–polynomial model was slightly lower than that of the Park–Gauss model, so the optimized Park–polynomial model was in better agreement with the measured wake.

## Conclusions

In this study, a linear correction of the SS model is proposed based on the RMSE of the SS model increasing linearly with the wake development. Two correction factors are fitted based on wind tunnel experimental data: *α* = 0.15 and *β* = 1.2 for the Park–Gauss model and *α* = 0.16 and *β* = 1.3 for the Park–polynomial model. Comparisons of the modified model with the SS model reveal that the 2D distribution of the full wake of the downstream wind turbine can be well simulated despite the wake interaction not being fully understood.

This study presents a modification of the wake superposition method, which can be utilized with the predicted full-wake model of downstream wind turbines. However, intensive efforts are still needed. The two coefficients of the linear correction require predetermining the flow field data under a typical arrangement. The accuracy of the mixed flow field strongly depends on* α* and *β*, and although the range of their values is given, generalization will be the focus of future work.

## Supplementary Information

Supplementary Information.

## Data Availability

All data generated or analyzed during this study are included in this published article and its Supplementary Information files.
